# Evolutionary foundations of social identity

**DOI:** 10.1098/rstb.2025.0157

**Published:** 2026-11-10

**Authors:** Leon Li, Sebastian Grueneisen, Michael Tomasello

**Affiliations:** ^1^ Faculty of Education, Leipzig University, Leipzig, Germany; ^2^ Department of Psychology and Neuroscience, Duke University, Durham, NC, USA

**Keywords:** groups, social psychology, shared agency, collective agency, moral identity

## Abstract

Social identity theory provides a rich description of how humans relate to various kinds of social groups, but no substantive account of why human psychology evolved to be so group-minded in the first place. We propose that the evolutionarily original form of social identity was one's moral identity as a cooperative group member. For ancient humans, survival depended on being an assimilated member of a cultural group, which, in turn, competed against other groups. In this context, humans evolved skills and motivations for engaging in the collective agency of a cultural group, especially the self-reflective capacity to adopt a social identity (i.e. to construe oneself as a member of a ‘we’) and the concomitant self-regulatory motivation to maintain a positive social identity (i.e. to strive for ‘I’, the individual, to meet the standards that ‘we’ uphold). In this evolutionary proposal, behavioural effects of group identity, inferences of common knowledge and moral emotions are all manifestations of the function of social identity as a self-regulatory system for maintaining one's good standing as a cooperative group member.

This article is part of the discussion meeting issue ‘From being to identity: analysis and synthesis of the self’.

## 1. Introduction

Social identity, or self-conception as a member of a group, has long been a prominent focus of cultural discourse as well as social psychological research [1]. Beginning with social identity theory [2], extensive research in social psychology has explored why individuals self-identify with groups, focusing on the proximate *psychological* level of explanation. This research has provided a rich description of the diverse proximate motivations that lead individuals to adopt a social identity, such as the needs for positive self-regard, distinctiveness, meaning and belonging [3,4]. However, classical accounts of social identity neglect the question of why individuals self-identify with groups on the *evolutionary* level of explanation, that is, why humans evolved to be so group-minded in the first place.

Modern humans construct complex social identities based on diverse features such as race, ethnicity, gender, nationality, occupation and social class. Although these features are significant aspects of modern life and have been the focus of much research, they were probably not core elements of social identity in its evolutionarily original form. As noted by Kinzler *et al.* [5, p. 625], race and ethnicity were not informative for most of human history because ‘neighboring groups of humans in ancient times did not likely look different in terms of physiognomy or skin colour’. While gender played a role in intragroup divisions of labour, it was not informative for intergroup differentiation. Nationality, occupation and social class are modern constructs that do not map onto the small-scale hunter-gatherer lifeways that comprised most of human history [6].

We propose that the evolutionarily original form of social identity was one's *moral identity* as a cooperative member of one's cultural group. If social identity is defined as self-conception as a member of a group [1], then, in order to determine the evolutionarily prototypical social identity, one must determine the evolutionarily prototypical group with which it was important for individuals to identify. This group was the *cultural group*—the large-scale bands of mutually acquainted tribes united by common language, customs, artefacts and so on—which we hypothesize emerged around the onset of the Late Pleistocene (around 130 kya). The moral dimension is of unique importance, as individuals' identification with the values and norms of the group—both following them and enforcing them on others—is what sustains group coherence.

In the following sections, we expand on this hypothesis. First, we elaborate on our evolutionary claim that the cultural group was the evolutionarily prototypical group. We outline a natural history of human evolution up to the point at which cultural groups acquired their critical significance as evolutionary selection pressures. Next, we bolster our psychological claim that humans are psychologically disposed to form a moral identity by reviewing evidence of its early manifestations in children.

## 2. Evolution of social identity

The natural starting point for discussing the evolution of uniquely human capacities, such as social identity, is the last common ancestor (LCA) of humans and other great apes. Extant great apes may serve as a model for the LCA because the capacities found in common across extant great ape species are more likely to have descended from a common ancestor than to have evolved independently multiple times in a short time frame [7]. Extant great apes exhibit diverse forms of instrumental rationality, such as planning for future goals, making causal and logical inferences, and self-monitoring uncertainty [8–10].

Therefore, the LCA probably had a relatively sophisticated kind of *individual agency*. By individual agency, we mean self-regulation for individual goal pursuit by means of *feedback control systems* such as self-monitoring and self-control. However, humans diverged from other great apes in their ability to engage in not only individual agency but also *shared agency*. By shared agency, we again mean self-regulation for goal pursuit, but in this case, the goal being pursued is a *joint goal* shared by multiple individuals and the mechanisms of self-regulation include *interpersonal* feedback control systems (whereby individuals regulate each other's behaviours), not just *intrapersonal* feedback control systems (whereby an individual only regulates his or her own behaviour).

Effectively, humans combined their individual ‘I-level’ agencies into shared ‘we-level’ agencies capable of ‘we-level’ goal pursuit. Humans became able to think and act from the perspective of a ‘we’: what ‘we’ want to do, how ‘we’ plan to do it and how ‘we’ regulate ourselves and each other in the service of joint goals [11,12]. While great apes display some apparently group-based behaviours, such as group border patrols or grooming of familiar others, such behaviours lack the interpersonal feedback control systems characteristic of human shared agency, as great apes do not appear to normatively expect others to engage in such behaviours or enforce ‘punishment’ against those who do not engage in them. Moreover, although great apes display certain mentalistic and behavioural foundations that are indeed necessary for engaging in joint action (e.g. gaze following and considering others' perceptual states, both necessary steps towards establishing joint attention; [13,14]), they do not appear to *coordinate* their mental states and actions in pursuit of joint goals, as humans do from early ages (for a review, see [15]).

### (a) Evolution of joint agency

We propose that the human capacity for shared agency evolved in two steps: *joint agency* and *collective agency*. The first step, the evolution of joint agency, occurred approximately 2 million years ago as early humans began engaging in collaborative foraging, such as working in groups to hunt or scavenge large game that individuals could not secure alone [16]. The shift from an individualistic to a collaborative foraging strategy may have been prompted by ecological changes, such as variations in climate that led to the expansion of terrestrial monkeys who competed with early humans for individualistically foraged food sources [7].

In game-theoretic terms, collaborative foraging aligns with the incentives of a *stag hunt* game. Rather than obtaining smaller pay-offs (hares) through individualistic action, actors are better off obtaining larger pay-offs (stags) through joint action. Great apes may be characterized as following a ‘hare-oriented’ strategy, with their reliance on individualistically gathered food sources such as leaves and fruits. While some chimpanzee groups hunt monkeys, they do so as a multitude of separate agents, each pursuing its own individual goal, not as a shared agent pursuing a shared goal. They do not intentionally communicate to initiate or coordinate group hunts (which are instead initiated via a ‘leader–follower’ dynamic in which individuals see that others are already pursuing a monkey and take advantage of this situation to try to catch it for themselves), nor do they intentionally share the spoils at the end [17].

Humans, by contrast, may be characterized as following a ‘stag-oriented’ strategy, with their reliance on collaboratively obtained food sources. Over iterations of the stag hunt across early human history, individuals who were better at engaging in shared agency with others, i.e. at forming a functioning ‘we’ capable of coordinating on larger pay-offs, were advantaged and selected for [7,18]. Thus, collaborative foraging was a key catalyst in the evolution of early human joint agency.

During this first phase of human evolution, social identity, in the sense of self-conception as a group member, probably did not play a significant role. This is because individuals engaging in small-scale (i.e. dyadic or small-group) collaborative activities characteristic of early human joint agency knew their collaborative partners on a first-hand basis. Moreover, even if individuals identified to some degree with different ‘familial subdivisions’ within their tribe, all tribe members were still directly acquainted. Assessing whether others were members of one's in-group did not become an issue until the second phase of human evolution, when humans began assembling in larger bands in which one was *not* necessarily acquainted with every other in-group member [19]. We hypothesize that this second phase of human evolution overlapped with the onset of the Late Pleistocene (around 130 kya), given the genetic and archaeological evidence of increases in human population sizes during this period [20,21].

### (b) Evolution of collective agency

The impetus for this second phase of human evolution was none other than the success of the first phase. Increases in the number and size of groups, owing to the efficacy of the early human collaborative foraging strategy, entailed corresponding increases in the competition *between* groups over resources. This context of intergroup competition favoured groups whose members were more loyal, coordinated and capable of collective action—or, in agentic terms, groups that were better able to engage in collective agency and act as a collective ‘we’ [22–24]. To sustain collective agency, groups used *group-level feedback control systems* to regulate the behaviours of individual group members in the service of group-level goals. These group-level feedback control systems undoubtedly included things such as social norms, institutions, fictive kinships, narratives, worldviews and other social realities that are still operative in human groups today [6,25]. We propose that social identity was also one of the group-level feedback control systems sustaining collective agency.

Social identity was necessary for both group and individual functioning. Regarding group functioning, group members needed a way to recognize who belonged to the group, given that members of large bands were not all personally acquainted. Social identity markers, such as clothing, language and conformity to group norms, fulfilled this need. This, in turn, enabled groups to ensure that the spoils of collaboration accrued to norm-abiding group members (rather than to outsiders or norm violators, such as non-reciprocators), which was important for sustaining group cooperation in the long run [26]. Moreover, tangible markers of group identity were also necessary for group members to be able to rally together into large-scale coalitional alliances, e.g. for group defence against a common enemy [27].

Regarding individual functioning, individual survival depended on being accepted by one's group. Indeed, this was especially true in the context of intergroup competition. As noted by Baumeister & Leary [28, p. 499], there is a ‘severe competitive disadvantage of the lone individual confronting a group when both want the same resource. When other people are in groups, it is vital to belong to a group oneself’. Displays of social identity helped ensure one's continued acceptance by the group and one's survival. Thus, both group and individual selection pressures favoured the evolution of skills and motivations for maintaining a social identity.

These skills and motivations included the cognitive capacity to adopt a social identity in the first place—the self-reflective capacity to construe oneself as a member of a collective group, a collective ‘we’. Building on this capacity, humans also evolved the concomitant self-regulatory motivation to maintain a *positive* social identity. As a point of clarification, by *positive* social identity, we do not mean the positive differentiation of oneself in comparison to others (e.g. [29,30]) but rather one's positive fulfilment of group expectations. Because groups have normative expectations for how group members should think, act, dress and so on, group members are continually immersed in evaluative standards for what it means to be a ‘good’ group member. Accordingly, a motivational disposition to *want* to be a ‘good’ group member, i.e. to *want* to maintain a positive social identity, was selected for because it facilitated individuals' acceptance by the group.

### (c) Evolution of moral identity

To be clear, we are not suggesting that the evolved motivation to maintain a positive social identity is solely a matter of strategically wanting to maintain a positive *reputation* in the eyes of others. Instead, it is also a matter of genuinely wanting to fulfil one's *own* values in one's own eyes. This is because individuals are socialized intensely from early on to internalize their group's cultural worldview as their own; the way ‘we’ see the world becomes the way ‘I’ myself see the world [25]. Consequently, whenever one attends to any object of perception in the world, one's perception of that object is coloured by the epistemic-normative lens of one's cultural worldview [11]. This is also the case when the object of perception is one's own self. One perceives one's own self (the ‘me’) from the value-laden perspective of the group (the ‘we’). As this self-perception is inherently normative, involving an evaluation of whether one's own self is fulfilling the values and standards that it should be, we term it ‘moral identity’. Our usage of the term moral identity thus aligns with psychological and philosophical accounts that individuals seek to maintain a positive self-conception as someone whose behaviours are consistent with their values [31–33].

We have proposed that moral identity evolved as a result of two steps in the evolution of human agency. First, humans diverged from the individualistic agency of other great apes in becoming able to form a joint ‘we’ for collaborative foraging. Later, humans expanded their sense of ‘we’ to encompass their cultural in-group as a whole, at which point moral identity—the self-reflective capacity to identify as a group member and the concomitant self-regulatory motivation to behave as a ‘good’ group member—evolved as the prototypical social identity.

Another point of clarification is worth mentioning. Our hypothesis that humans evolved capacities and motivations for sustaining collective agencies and moral identities does not mean that such adaptations emerge ‘innately’ in ontogeny as a direct outcome of gene expression. Humans, with their highly plastic brains and slow periods of maturation [34], are exceptionally receptive to learning from the inputs of their species-typical niche. For humans, the species-typical niche is a cultural niche. As such, the specific forms of collective agency and moral identity that individuals develop depend on both their social learning of the inputs specific to their culture and the *kinds* of inputs offered by their culture, as determined by cultural evolution [23]. Neither of these processes (i.e. individual-level social learning or group-level cultural learning) is reducible to genetic bases alone. For example, on the scale of cultural evolution, adaptive cultural practices may propagate across groups simply owing to the fact that successful strategies attract imitators [35], without the need for a specific genetic basis for a given strategy. In the next section, we review the developmental evidence for the psychological claim that humans are psychologically disposed to form a moral identity.

## 3. Ontogeny of social identity

In ontogeny, as in evolution, moral identity emerges in two steps. Children first develop competencies for joint agency, which later scale up into competencies for collective agency. We review this shift in four key competencies relevant to moral identity: social self-regulation, prosociality, common ground and moral emotions (table 1).

**Table 1.** Four key competencies relevant to moral identity.

competency	joint agency		collective agency
social self-regulation:	joint commitments	→	social norms
prosociality:	helping partners	→	in-group favouritism
common ground:	personal	→	cultural
moral emotion:	personal guilt	→	collective guilt

### (a) Social self-regulation

The prototypical form of social self-regulation underlying joint agency is a *joint commitment*. Once you and I commit to an activity together, our commitment creates obligations for how we should act. We expect each other to play our respective roles in the activity, not to abandon the activity arbitrarily, and to respectfully ‘take leave’ when we want to exit the activity. By the age of 3 years, children understand joint commitments and their accompanying obligations. For instance, 3-year-olds are more likely to resist temptations to abandon an activity and are more likely to take leave when exiting an activity if they had made a commitment to do the activity with the partner than if they had not made a commitment [36,37]. Vasil & Tomasello [38] found that simply framing an activity as something ‘we’ are doing together, as opposed to something ‘you’ and ‘I’ are doing in parallel but separately, increases 3-year-olds' leave-taking when exiting an activity.

When reacting to partners who fail to play their role in a joint commitment, 3-year-olds protest more if the partner intentionally defects from the activity than if the partner is simply ignorant about how the activity works [39], and they also protest more if the partner abandons the activity abruptly than if the partner takes leave [40]. Altogether, these studies demonstrate how 3-year-olds already possess interpersonal and intrapersonal feedback control systems for engaging in joint commitments, such as protesting against uncooperative partners or delaying individual gratification for joint goals; see also [41,42].

Analogous to how joint agency is regulated by joint commitments, collective agency is regulated by *social norms*. A joint commitment refers to an agreement between social partners that ‘we’ (meaning the *specific* individuals in the immediate situation) agree to do something together in a certain way. A social norm also refers to an agreement between social partners, but one in which the sense of ‘we’ is scaled up to encompass not just the specific individuals in the immediate situation but rather all members of one's group, including members whom one may not even know. Essentially, a social norm exists when ‘we’ members of a group agree to do something together in a certain way. Children understand social norms by the age of 3 years, as evidenced by their willingness to not only follow norms but also enforce them and protest when others violate them [43].

Importantly, children also understand that following group norms is an integral part of identifying with a group. In a study by Schmidt *et al.* [44], 3-year-olds protested more against a violation of an in-group conventional norm if the violator was an in-group member as opposed to an out-group member, reflecting an understanding that the norms that ‘we’ endorse apply primarily to those who identify with the ‘we’ and not so much to outsiders. In a study by Li *et al.* [45], which focused on children's own adherence to in-group norms, 3-year-olds were invited to identify with a university in-group (‘We are Duke!’) and then set up a tea party. The 3-year-olds conformed more to a model's choices of tea party items when the choices were framed as in-group norms (e.g. ‘For tea parties at Duke, we always use this kind of cup’) versus personal preferences (e.g. ‘For my tea party today, I feel like using this cup’). Altogether, children's early-emerging understanding, observance and enforcement of social norms reflect a deep psychological disposition to maintain a positive moral identity as someone who respects and fulfils the norms of the group.

### (b) Prosociality

Children as young as 2 years old perform prosocial acts, such as helping and sharing [46]. By the age of 3 years, children show heightened prosociality towards collaborative partners with whom they have formed a joint agency. In a study by Hamann *et al*. [47], pairs of 3-year-olds retrieved rewards from a pulling apparatus through either individual or joint action. The 3-year-olds were more likely to equally share the obtained resources if their partner's help had been necessary for retrieving the resources, compared to if they had retrieved the resources individually. A follow-up study found that after 3-year-olds had already obtained their own reward in a collaborative activity, they were still willing to keep collaborating until their partner also obtained a reward [48]. Young children even cheat in games specifically to benefit collaborative partners or previous benefactors [49,50], revealing the force with which collaborative interactions impact children's prosocial motivations.

With age, children's prosocial tendencies appear to scale up from being directed towards specific collaborative partners to being directed towards in-group members in general. As strikingly demonstrated in the *minimal group paradigm*, in which individuals are assigned to groups that differ only along arbitrary features such as clothing colour, children from 4 to 5 years of age show diverse forms of in-group favouritism, such as greater in-group liking, helping, sharing and loyalty when keeping a secret, despite the arbitrariness of the in-group identity [51–53]. As proposed by Dunham [54, p. 786], such in-group-favouring tendencies may reflect ‘an evolved system of coalitional reasoning’. In agentic terms, they may reflect an evolved system for engaging in collective agency with in-group members. In any case, they show that adopting a social identity leads children to behave prosocially towards others who share the same identity.

### (c) Common ground

To collaborate effectively in a joint agency, partners need to have common ground—mutual knowledge about ‘what we both know that we both know’ [55,56]. By the age of 3 years, and increasingly throughout the later preschool years, children track how much common ground they share with a partner when discussing what to do and justifying their decisions [57,58], as well as when coordinating their decisions without explicit communication [59,60]. By the age of 3 years, children also understand that groups are constituted by *mutual intentions*—the common ground consensus among group members that ‘we’ all want each other to be part of the same group [61,62].

With increasing age, children come to realize that they share not only *personal* common ground with specific others based on shared direct experience, but also *cultural* common ground with in-group members in general based on shared cultural experiences [63]. Soley [64] found that 5-year-olds expect greater common ground about music, an important form of culture, among members of the same group than among members of different groups. In another study, Soley & Aldan [65] found that 5-year-olds expect greater common ground about music among individuals who speak the same language (an honest signal of group enculturation) than among individuals of the same gender (a non-coalitional identity). A third study by Karadağ & Soley [66] found that 5-year-olds prefer to teach conventional norms to in-group members as opposed to out-group members.

Overall, cultural common ground is a necessary condition for effective collaboration at the level of large groups. It allows in-group members to coordinate with each other, even when personal common ground is sparse. By the age of 5 years, children understand that cultural common ground is tied to group identity and are motivated to help fellow in-group members acquire this cultural knowledge.

### (d) Moral emotion

An important competency for engaging in joint agency is relationship repair. Joint activities do not always go according to plan, and sometimes one's own self is to blame. In such cases, it becomes incumbent upon oneself to try to make amends and repair the relationship. The prototypical moral emotion befitting this situation is *guilt*, which is operative by the age of 3 years. In a study by Vaish *et al.* [67], 3-year-olds were led to believe that they had harmed another person's property. As a result, the 3-year-olds displayed signs of guilt, such as taking responsibility for the act and trying to make reparations. The fact that humans feel sincerely guilty about wrongdoing (at least some of the time) is evidence, in our view, that individuals genuinely internalize the viewpoint of their group (i.e. ‘how *we* see me’) and are not solely driven by strategic reputational concerns (i.e. ‘how *others* see me’).

By the age of 5 years, children display a particularly striking effect of social identity. As shown by Over *et al.* [68], 5-year-olds feel *collective guilt*—guilt about transgressions that were committed not by oneself but by fellow in-group members. In their study, 5-year-olds displayed more nonverbal signs of guilt and were more willing to take reparative action following a transgression committed by an in-group member versus an out-group member. Thus, adopting a social identity as a member of a group can have such a profound effect on children that they even feel responsible for the actions of fellow group members.

### (e) Justification

Another phenomenon worth noting, although it does not directly map onto our proposed two-step sequence from joint to collective agency, is the use of *justifications* to preserve one's moral identity. We briefly review the developmental research on justifications, as they are an important part of how individuals construct moral identities and defend them in the eyes of themselves and others. Like guilt displays, justifications mitigate the negative impact of one's own failings. From 3 to 5 years of age, children progress in their reason-giving and justification abilities, and are eventually able to use sophisticated discourse strategies such as counter-arguments, indirect evidence and meta-talk [69–71].

Kanngiesser *et al.* [72] examined children's ability to justify their own transgressions by enticing them to break a promise and then asking them what happened. At the age of 3 years, children provided more relevant justifications if they had a prosocial reason for breaking the promise (e.g. they had been asked to help someone) than if they only had a selfish reason (e.g. they were playing with a toy), reflecting an early-emerging understanding that prosocial reasons are more acceptable than selfish ones. Children from 3 to 5 years of age also prioritize prosocial over selfish justifications when evaluating others' reasons for promise-breaking [73].

Over ontogeny and into adulthood, individuals become more skilful at deploying justifications to protect their moral identity in the eyes of both themselves and others. While individuals desire, on the one hand, to be seen by both themselves and others as morally upright, they also desire, on the other hand, to reap the benefits of unethical acts. Self-serving justifications and rationalization techniques, e.g. *moral disengagement* (i.e. downplaying an unethical action as ‘not so bad’) or *deliberate ignorance* (i.e. choosing not to know about the consequences of one's actions), give individuals justificatory ‘wiggle room’ to act unethically while maintaining a positive moral identity [74–76].

## 4. Conclusion

Our theory would benefit from empirical testing. For example, we have claimed that children's norm-following is not reducible to a desire for a positive reputation (i.e. ‘how others see me’) but also reflects a genuine internalization of the group's legitimate evaluative viewpoint (i.e. ‘how we see me’). Research has found that children act more prosocially when observed by others, reflecting reputational concerns [77,78]; however, more studies are needed to investigate how children act when unobserved, reflecting their internalized values. As one example, Engelmann *et al.* [79] found that 5-year-olds sometimes do the right thing and share with a needy recipient, even if no one would know about their generous act. Moreover, we have claimed that competencies for collective agency are ‘scaled-up’ versions of earlier-emerging competencies for joint agency. This claim could be tested through longitudinal studies on the emergence of different competencies for joint and collective agency.

Several future directions of research could be built on the present proposal. For instance, what are additional competencies for collective agency beyond the four that we have discussed? What are the impacts of individual difference variables (e.g. temperament, executive function, cultural background) on children's competencies for collective agency and moral identity? Two variables whose effects on moral identity would be interesting to explore are autobiographical memory and theory of mind. Autobiographical memory, the ability to integrate multiple memories into an overarching self-conception [80], seems to be necessary for sustaining a moral identity over time. Theory of mind, the ability to represent others' mental states, is closely associated with the ability to represent one's own mental states [81], which, in turn, probably plays a pivotal role in the construction of the self-concept. Theory of mind also enables one to predict how others will react to one's own actions. An awareness that others are more likely to react predictably when one's own actions are predictable (e.g. in line with social norms), coupled with the fact that predictable information is metabolically preferable (i.e. less costly) to process than unpredictable information [82], may lead individuals to have an intrinsic motivation to follow social norms and maintain a positive social identity. The metabolic bases of individuals' self-regulatory attempts to maintain their social identities would be interesting to explore in future research.

In conclusion, we propose that humans have an evolved psychological disposition, evident in young children, to adopt and maintain a positive moral identity as a cooperative group member. The skills and motivations to adopt and maintain a moral identity are part of the suite of psychological competencies that humans evolved for engaging in collective agency, building on evolutionarily and ontogenetically earlier capacities for engaging in joint agency. Our proposal may offer a novel evolutionary lens for interpreting the diverse and profound effects of social identity on human thinking and behaviour.

## Data Availability

This article has no additional data.

## Theme

One contribution of 17 to a discussion meeting issue ‘From being to identity: analysis and synthesis of the self’.
